# Comparative Study on the Mechanical Behavior of Flax and Glass Fiber Multiaxial Fabric-Reinforced Epoxy Composites

**DOI:** 10.3390/ma18194469

**Published:** 2025-09-25

**Authors:** Carsten Uthemann, Thomas Gries

**Affiliations:** Institut für Textiltechnik (ITA) of RWTH Aachen University, 52074 Aachen, Germany

**Keywords:** flax fiber, glass fiber, epoxy composites, multiaxial non-crimp fabric, sustainability, mechanical properties

## Abstract

This study presents a comparative investigation of the mechanical performance of epoxy-based composites reinforced with ±45° multiaxial non-crimp fabrics (NCFs) made from natural flax fibers and conventional glass fibers. Flax fibers, despite their attractive sustainability profile and favorable specific mechanical properties, are typically processed into twisted yarns for textile reinforcement, which compromises fiber alignment and reduces composite performance. A novel yarn-free flax NCF was developed using false twist stabilization of aligned slivers to eliminate the negative effects of yarn twist. Composite laminates were manufactured via vacuum-assisted resin infusion (VARI) under identical processing conditions for both flax- and glass-based reinforcements and tested for tensile, compressive, and flexural behavior. The results show that, although glass fiber composites exhibit superior absolute strength and stiffness, flax-based NCF composites offer competitive specific properties and benefit significantly from improved fiber alignment compared to yarn-based variants. This work provides a systematic comparison under standardized conditions and confirms the mechanical feasibility of flax NCFs for semi-structural lightweight applications.

## 1. Introduction

Fiber-reinforced composites (FRCs) are extensively used in automotive, aerospace, and sports applications due to their high specific strength and stiffness, enabling substantial weight savings over traditional materials. Among them, glass fiber-reinforced composites (GFRCs) are dominant due to their excellent mechanical performance and cost-effectiveness [1]. However, the increasing need for sustainable and renewable materials has driven research toward natural fiber-reinforced composites (NFRCs), particularly those reinforced with bast fibers such as flax, hemp, and jute [2,3,4]. Flax fibers are among the most studied natural reinforcements due to their high tensile stiffness, low density, biodegradability, and relatively low energy requirement during production [5]. The intrinsic tensile modulus of flax can reach up to 80 GPa, with tensile strength exceeding 1000 MPa under ideal conditions [6]. However, these values are rarely retained in composite form, primarily due to the influence of fiber processing and architecture [7].

One critical limitation of flax fiber-reinforced composites lies in textile processing. The transformation of technical fibers into spun yarns involves twist insertion, which leads to fiber misalignment and increased crimp. Both factors reduce the effective load-bearing capacity of the fibers in composite matrices [8,9]. Pickering et al. [3] and Rayyaan et al. [7] highlight that avoiding twisting and minimizing processing damage are key factors to transferring the intrinsic fiber properties into the composite part. Shah [10] emphasized that the deviation of fiber orientation from the loading direction has a detrimental impact on tensile properties. Moreover, crimp and waviness—typical in woven fabrics—introduce stress concentrations that reduce compressive and shear strength [11].

To mitigate issues such as fiber undulation and misalignment, non-crimp fabrics (NCFs) directly produced from natural fiber slivers have emerged as a promising solution. NCFs consist of aligned fiber layers held together by stitching yarns, thereby minimizing crimp and making better use of the intrinsic fiber properties. Studies on NCFs have demonstrated significantly improved mechanical performance compared to woven equivalents [7,12].

However, applying this concept to flax slivers—loosely assembled, untwisted bundles of parallel fibers obtained during carding or combing—remains challenging due to their lower cohesion and reduced processing robustness compared to yarns or synthetic rovings. A fully industrializable multiaxial NCF concept based exclusively on untwisted flax slivers has yet to be realized. Furthermore, despite the potential of such architectures, systematic mechanical comparisons between flax and glass fiber NCF composites—particularly under equivalent fiber orientations and matrix systems—are scarce. Most comparative studies differ in fabric architecture, resin type, or manufacturing process, making direct benchmarking difficult [13]. Moreover, processing differences, especially in compaction behavior and achievable fiber volume content, strongly influence mechanical results.

The objective of this study is therefore to close this research gap by developing a ±45° flax NCF using a novel yarn-free production process based on false twist stabilization. The resulting flax/epoxy laminates are mechanically characterized and directly benchmarked against reference glass NCF composites manufactured under identical conditions, ensuring that observed differences can be attributed to the reinforcement architecture and fiber type rather than to variations in processing. This targeted approach highlights both the opportunities and current limitations of flax NCFs, providing a clearer basis for future material optimization.

## 2. Materials and Methods

### 2.1. Textile Reinforcements

This study evaluates three types of NCFs (Table 1). A commercially available E-glass NCF made from continuous rovings serves as the mechanical performance benchmark. A second NCF, produced from twisted flax yarns, represents the current industrial standard for NFRCs. The third material is a flax NCF manufactured from untwisted flax slivers using a novel, yarn-free processing route described in Section 2.2.

### 2.2. Production of the Sliver-Based Flax NCF

**Figure 1:**
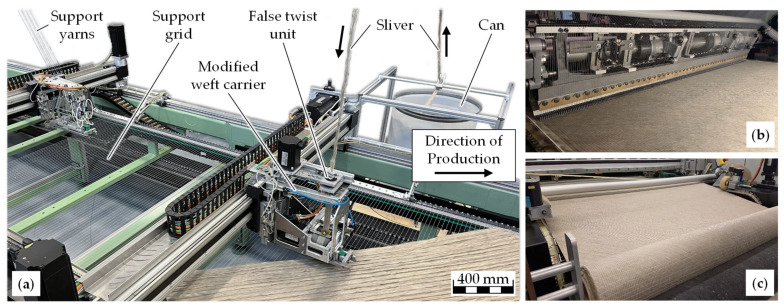
(**a**) Production setup for sliver-based flax NCF with false-twist feeding unit; (**b**) NCF after stitching; (**c**) wound NCF roll. Adapted from [14].

The sliver-based flax NCF was manufactured on a Copcentra MAX 3 CNC multi-axial warp-knitting machine (LIBA Maschinenfabrik GmbH, Naila, Germany; now part of KARL MAYER Holding SE & Co. KG, Obertshausen, Germany) that was modified at the Institut für Textiltechnik (ITA) of RWTH Aachen University, Aachen, Germany. A detailed description of the modification is provided in [14]. The setup is shown in Figure 1.

The process begins with the sliver feeding unit, which ensures continuous, twist-free delivery of flax slivers from a rotating can. These slivers pass through a false-twist unit, where they are temporarily twisted to provide consolidation and improved handling during feeding. The consolidated slivers are then transferred to the weft carrier, which lays them at ±45° orientations onto a supporting polyester grid to achieve the desired reinforcement architecture. Subsequently, the layers are secured in place by the stitching unit using polyester stitching yarns. Finally, the completed NCF is collected by the winding unit, where it is wound onto rolls for storage and further processing. An overview is given in Table 2.

### 2.3. Fiber Orientation

**Figure 2:**
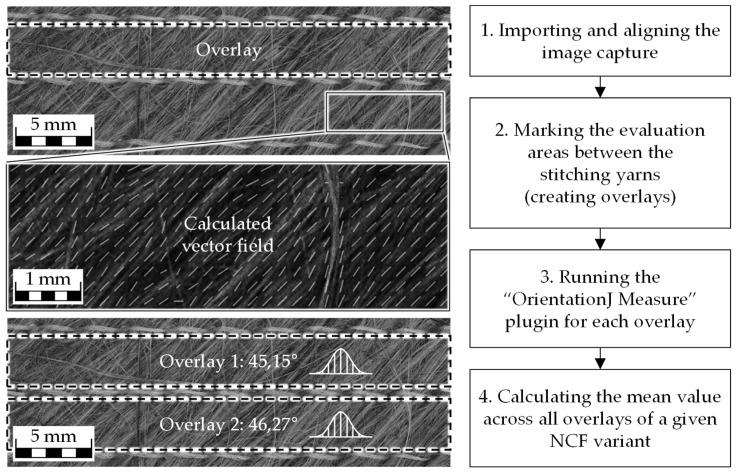
Procedure for determining the average local fiber orientation in NCF samples using the ImageJ plugin OrientationJ. Images were analyzed in grayscale, since orientation was derived from brightness gradients rather than color information. The images show the surface of a single ±45° NCF ply analyzed for orientation. Adapted from [14].

Load-appropriate alignment of reinforcing fibers is one of the most critical factors for achieving high mechanical performance in FRCs. A well-defined fiber orientation in the NCF is therefore of particular importance. At present, there is no standardized test method for determining the fiber orientation of textile reinforcements. In this study, the local fiber orientation is assessed by analyzing images of the NCF surfaces. Image evaluation is performed using the open-source software ImageJ 1.54d in combination with the OrientationJ plugin 2.05 [15,16,17]. The local orientation is calculated from brightness gradients in grayscale images based on structure tensors. A detailed description of this method is provided in [15,16,18]. The used test procedure is illustrated in Figure 2. The alignment of the images was carried out according to the stitching yarn paths in the direction of NCF production (0°). The tests were conducted at room temperature.

In the present study, reproducibility was addressed by analyzing 24 overlays per material variant, which reduces operator bias and provides robust mean values. Comparable applications of the OrientationJ plugin have been reported in a variety of fibrous systems, including hemp fiber mats [19], short fiber composites validated against µCT [20], electro-spun materials [21], and fibrillar biological structures [22,23]. These studies support the reliability of the OrientationJ approach for quantifying fiber orientation, although a systematic inter-operator reproducibility assessment remains an important task for future work.

### 2.4. Composite Manufacturing

All three NCF types were processed into composite laminates using VARI. The epoxy system consists of EPIKOTE™ RIM R135 and the hardener EPIKURE™ RIM H137, both supplied by Hexion Inc., Columbus, OH, USA. The components were mixed at a weight ratio of 100:30. The system offers a pot life of approximately 300 min and cures to a density of around 1.15 g/cm^3^. For tensile and compressive testing, end tabs were bonded using the two-component adhesive ARALDITE^®^ 2011 GB from Huntsman Corp., The Woodlands, TX, USA. Test specimens were cut using a water-cooled circular saw type K680 from MAIKO Engineering GmbH, Braunschweig, Germany. The processing sequence for manufacturing the coupon specimens was as follows:

1. Lay-up of four NCF plies in a [0/90]_4_ stacking sequence, aligning the ±45° NCF layers such that their principal fiber orientations corresponded to 0° and 90°;
2. Drying in a convection chamber furnace N 60/65SHA (Nabertherm GmbH, Lilienthal, Germany) at 80 °C for 30 min;
3. Integration into VARI setup, including peel ply, flow media, and vacuum bagging;
4. Resin infusion at ambient temperature under a vacuum of approximately −0.9 bar;
5. Bonding of end tabs for tensile and compressive testing;
6. Post-curing in a convection chamber furnace N 60/65SHA (Nabertherm GmbH, Lilienthal, Germany) at 80 °C for 10 h;
7. Application of strain gauges for compressive testing.

All laminates were manufactured under identical conditions to reflect process-realistic constraints and enable a like-for-like comparison of the three NCF architectures. Under these conditions, continuous E-glass rovings exhibit higher compaction efficiency than flax-based reinforcements. We therefore frame the comparison as process-controlled, acknowledging that alternative processes with higher compaction pressures (e.g., RTM) may increase the achievable FVC of flax laminates but fall outside the scope of this VARI-focused study.

### 2.5. Mechanical Testing

To evaluate the mechanical performance of the composite laminates, standardized tensile, compressive and flexural tests were conducted.

#### 2.5.1. Tensile Testing

Tensile strength and stiffness of the three material variants were determined in accordance with DIN EN ISO 527-4 [24] using type 3 specimens equipped with glass fiber-reinforced plastic end tabs. Tests were carried out on a Z100 universal testing machine (ZwickRoell GmbH & Co. KG, Ulm, Germany) equipped with a 100 kN load cell and wedge grips. Deformation was measured using an RTSS video extensometer (LIMESS Messtechnik und Software GmbH, Krefeld, Germany). The samples were subjected to a preload of 100 N. The testing speed was 2 mm/min. This crosshead speed corresponds to the recommendation in DIN EN ISO 527-4 for tensile testing of orthotropic fiber-reinforced composites.

#### 2.5.2. Compressive Testing

Compressive properties were determined in accordance with DIN EN ISO 14126 [25], method 2, using type B1 specimens with bonded glass fiber-reinforced plastic end tabs. This method applies a combination of shear stress and end-load-induced compression to the central portion of the specimen. The calibrated specimen length (L, i.e., the gauge section between the tabs) was determined according to the specifications given in ISO 14126 for type B1 specimens.

**Figure 3:**
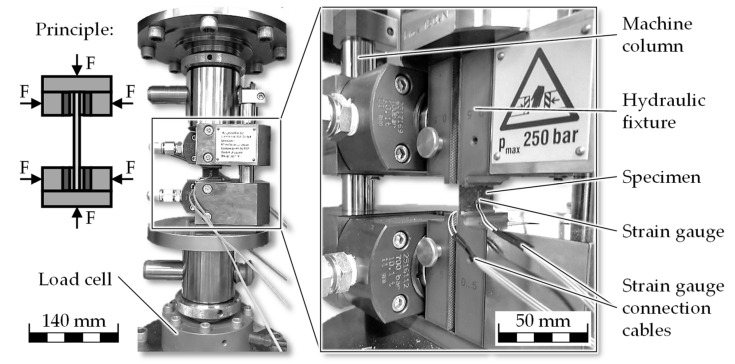
Compression testing setup using combined end-loading and shear loading according to DIN EN ISO 14126 (method 2, type B1 specimens). Adapted from [14].

Testing was performed on a Z100 universal testing machine (ZwickRoell GmbH & Co. KG, Ulm, Germany) equipped with a 100 kN load cell and an HCCF hydraulic compression fixture for composite materials (ZwickRoell GmbH & Co. KG, Ulm, Germany). The fixture provides lateral support and thus prevents premature buckling of the specimen during loading. A representative specimen mounted in the fixture is shown in Figure 3.

Longitudinal strain was recorded using strain gauges of type FLAB-3-11-1LJCT-F (Tokyo Measuring Instruments Laboratory Co., Ltd., Tokyo, Japan), applied symmetrically on both sides of the specimen. No preload was applied during testing. The crosshead speed was set to 1 mm/min, in line with ISO 14126 recommendations, and specimens were loaded until failure. A drop in force of 30% relative to the maximum recorded load was defined as the termination criterion. Specimens exhibiting clamp failure or a deflection exceeding 10% were considered invalid. Deflection was calculated from the compressive strain values recorded by the strain gauges mounted on the front and rear faces of the specimen.

#### 2.5.3. Flexural Testing

Flexural properties were determined using three-point bending tests in accordance with DIN EN ISO 14125 [26]. The tested materials were assigned to material class III. Testing was performed on a type 1455 universal testing machine (ZwickRoell GmbH & Co. KG, Ulm, Germany) equipped with a 1 kN load cell. The supports and the bending punch each had a radius of 5 mm. Specimens were positioned such that the outermost fiber layer on the tension side faced downward. Deflection was recorded via the crosshead displacement of the testing machine. Testing was terminated upon visible specimen failure or a drop in force of 30% relative to the maximum load. Relevant specimen dimensions and test setup parameters are listed in Table 3. The test speed was 2 mm/min. This speed corresponds to the recommendation of DIN EN ISO 14125 for class III composite specimens. After testing, failure modes were determined based on visual inspection of the fracture surfaces.

### 2.6. Fiber Volume Content and Composite Density

The fiber volume content (FVC) of the laminates was calculated based on the total plate mass, the mass of the NCF reinforcements, and the densities of both the epoxy matrix and the reinforcing fibers. The composite density ρ_Vi_ was derived analytically using the determined global fiber volume content φ_Fi_, the matrix density ρ_Mi_, and the respective fiber density ρ_Fi_, according to Equation (1). Porosity was not considered in this calculation.

ρ_Vi_ = φ_Fi_ · ρ_Fi_ + (1 − φ_Fi_) · ρ_Mi_ (1)

## 3. Results

### 3.1. Local Fiber Orientation

Table 4 summarizes the average local fiber orientation and the absolute deviation from the target angle of 45° for all investigated NCF variants. For the flax fiber NCFs, individual measurements deviate by up to 6.6° from the target orientation. In the yarn-based flax NCF, the measured values represent the orientation of the yarns, whereas in the sliver-based flax NCF, the average orientation of the individual fibers is determined. Consequently, variations in fiber alignment within the fiber sliver are also reflected in the results. In the roving-based glass NCF, fluctuations are comparatively small, at ~1.4°.

### 3.2. Tensile Properties

Tensile test data are summarized in Table 5. The roving-based glass fiber NCF exhibits the highest tensile performance, reaching 527.38 MPa in strength and 25.61 GPa in modulus. Both flax-based variants display lower absolute values, as expected due to the lower intrinsic stiffness and strength of flax fibers compared to E-glass. Among the flax composites, the sliver-based NCF records 126.47 MPa in strength and 10.46 GPa in modulus, representing a ~6% strength gain and ~27% modulus increase relative to the yarn-based variant (118.87 MPa; 8.26 GPa).

**Figure 4:**
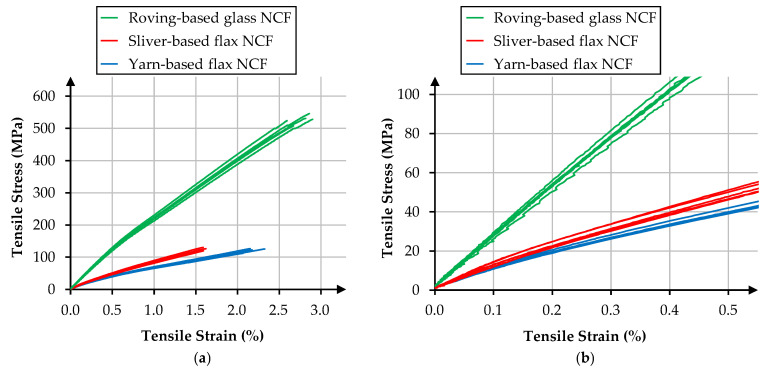
Tensile stress–strain curves of NCF composites: (**a**) complete curves up to failure; (**b**) detailed view of the low-strain region (0–0.5% strain) highlighting differences in initial stiffness.

The higher modulus likely results from the improved alignment of individual fibers within the slivers in the load direction, enabling the fibers to bear stresses even at low strain levels (Figure 4b). In contrast, the yarns possess a greater crimp reserve, which diminishes tensile stiffness.

### 3.3. Compressive Properties

**Figure 5:**
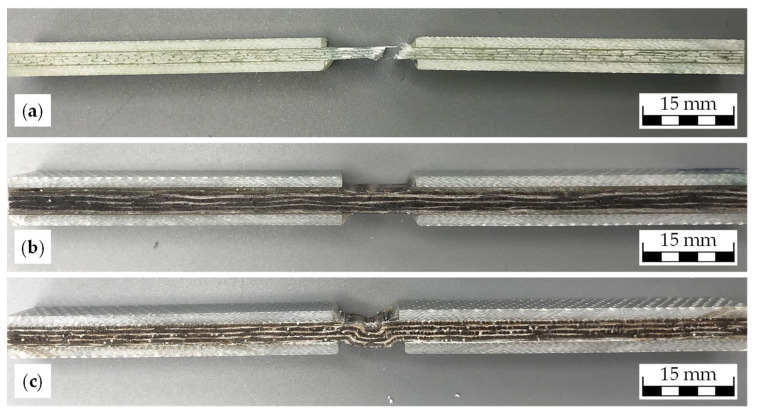
Representative composite specimens after compression testing: (**a**) roving-based glass NCF, (**b**) sliver-based flax NCF, and (**c**) yarn-based flax NCF.

The compressive performance results, presented in Table 6, follow a similar trend to tensile properties. The glass fiber NCF achieves 437.62 MPa in strength and 26.98 GPa in modulus. The sliver-based flax NCF records 89.64 MPa in strength and 9.76 GPa in modulus, compared to 89.24 MPa and 8.62 GPa for the yarn-based flax NCF. While strength is essentially identical between the two flax variants, the sliver-based version delivers ~13% higher modulus, again pointing to the stiffness benefits of improved fiber alignment and reduced undulation. Representative images of the tested specimens are shown in Figure 5.

### 3.4. Flexural Properties

Flexural test results are given in Table 7. The roving-based glass fiber NCF leads with 640.30 MPa in strength and 13.31 GPa in modulus. The sliver-based flax NCF achieves 148.59 MPa in strength and 6.58 GPa in modulus, compared to 144.19 MPa and 5.35 GPa for the yarn-based flax NCF. This translates to ~3% higher strength and ~23% higher modulus for the sliver-based variant, reinforcing the positive effect of better fiber alignment under combined tensile and compressive stresses.

**Figure 6:**
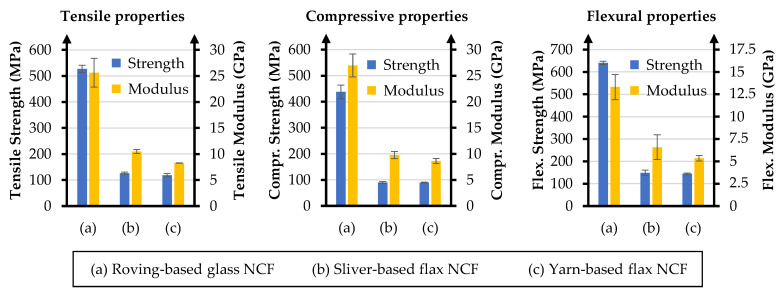
Comparison of overall mechanical properties of the investigated NCF composites. Values include tensile, compressive, and flexural strength and modulus. Data from Table 5, Table 6 and Table 7.

To facilitate direct comparison across all loading modes, the tensile, compressive, and flexural properties of the three NCF laminates are summarized in Figure 6. This overview highlights the superior absolute performance of glass fiber composites and the consistent stiffness advantage of the sliver-based flax NCF compared to the yarn-based variant.

### 3.5. Specific Mechanical Properties

The glass and flax laminates were produced under identical conditions, which led to different FVCs due to the intrinsic compaction behavior of the respective reinforcements; this process-driven difference is reflected in the density and specific property results. The measured and calculated densities, along with fiber volume contents, are summarized in Table 8. The two flax-based composites share the same composite density (1.23 g/cm^3^) due to similar FVC (~31%) and identical constituent densities.

Specific mechanical properties are shown in Table 9. Across all loading modes, the sliver-based flax NCF outperforms the yarn-based variant, with the largest relative improvements seen in the specific moduli.Relative to the glass fiber benchmark, the specific tensile modulus amounts to ~62%, while the specific flexural modulus reaches ~75% of the corresponding reference values.

## 4. Discussion

### 4.1. Influence of Fiber Architecture on Stiffness and Strength

As shown in Table 4, the measured local fiber orientations of the investigated NCFs are within ±2° of the target 45° for all variants, with slightly larger deviations observed in the flax-based materials. The sliver-based flax NCF shows an average deviation of 2.05°, compared to 1.45° for the yarn-based variant and 1.43° for the roving-based glass NCF. These small angular differences, although modest in absolute terms, can influence the efficiency of load transfer along the fiber axis, particularly in stiffness-driven loading modes. The improved alignment of the sliver-based flax NCF, combined with the absence of yarn twist, contributes to its consistently higher moduli observed in tensile, compressive, and flexural tests.

The comparative data presented in Section 3 clearly show that the textile architecture of the NCF reinforcement plays a decisive role in the mechanical performance of the composites. Across all loading modes, the sliver-based flax NCF consistently outperforms the yarn-based variant in terms of modulus, with relative gains of +27% for tensile, +13% for compressive, and +23% for bending properties. These results confirm that improved fiber alignment, achieved by eliminating yarn twist and reducing undulation, leads to more efficient stress transfer along the fiber axis. Similar conclusions were reported by Rayyaan et al. [7] and Ueki et al. [27], who found that even modest reductions in misalignment can translate into substantial stiffness improvements in natural fiber laminates.

The underlying mechanism is straightforward: yarn twist introduces helical fiber paths within the yarn cross-section, meaning that only the axial component of the fiber stress contributes to load bearing in the intended direction. In addition, yarn undulation within the NCF layer causes local fiber bending and induces shear stresses in the matrix, both of which lower the effective modulus. In the sliver-based NCF, untwisted fiber bundles are held in a near-linear configuration, maximizing axial load sharing and reducing matrix-dominated regions.

Strength increases for the sliver-based flax NCF are more modest—+6% in tensile strength and +3% in flexural strength—while compressive strength remains unchanged. This discrepancy between stiffness and strength gains is consistent with the findings of Yan et al. [28], who noted that ultimate strength in bast fiber composites is often governed by intrinsic fiber flaws, lumen geometry, weak links or imperfections introduced during fiber extraction and processing. Orientation improvements alone cannot fully overcome these microstructural constraints.

### 4.2. Compressive Behavior and Microbuckling Resistance

Under compressive loads, both flax-based variants achieve similar strengths (~89 MPa), but the sliver-based NCF exhibits a noticeably higher modulus. This higher compressive stiffness is indicative of reduced fiber waviness, which delays the onset of microbuckling. Bos et al. [29] demonstrated that fiber crimp is a primary trigger for kinking in flax fiber composites, and its reduction directly enhances the elastic response in compression.

To complement the mechanical data, representative images of the fracture surfaces of tested specimens were included (Figure 5). The glass fiber NCF (Figure 5a) failed abruptly through brittle crushing and splitting of the rovings, which is characteristic of synthetic fiber composites. The sliver-based flax NCF (Figure 5b) exhibited localized matrix shear cracks oriented at approximately 45° to the loading direction, consistent with shear-driven resin failure under compressive stress. Importantly, no distinct kink-band formation was observed, suggesting that the straighter fiber alignment in the sliver-based material effectively suppressed microbuckling. By contrast, the yarn-based flax NCF (Figure 5c) showed explicit kink-band formation and microbuckling within a wider deformation zone, reflecting the influence of residual fiber undulation from the yarn architecture.

These macroscopic observations help visualize the dominant fracture modes and support the interpretation that compressive strength in flax composites is limited primarily by matrix shear failure (sliver-based) or by fiber instability through kink-band formation (yarn-based). At the same time, compressive stiffness benefits directly from improved fiber alignment. While such observations are valuable, more detailed investigations using scanning electron microscopy (SEM) or computed tomography (CT) would provide deeper insights into fiber–matrix interactions and microstructural damage evolution. These analyses were not available for the present study but are considered an important direction for future work.

### 4.3. Flexural Response and Combined Loading Effects

Flexural tests, which involve tension on one face and compression on the opposite face of the laminate, highlight the structural advantage of the sliver-based architecture under combined loading. The +23% flexural modulus increase mirrors the tensile modulus gain, confirming that improved fiber orientation benefits both the tensile and compressive sides of the beam.

Flexural strength gains are smaller (~3%), reflecting the fact that stiffness benefits do not fully translate into ultimate load capacity. In bending, crack initiation can occur on either the tensile or compressive side, depending on the dominant flaw population. For bast fibers, relatively large lumens and variability in cell wall thickness are likely crack initiation points under tensile bending loads, whereas matrix shear failure governs the compressive side. Both mechanisms are only weakly influenced by improved fiber orientation.

### 4.4. Process-Controlled Comparison and Implications of FVC Differences

The present work benchmarks flax and glass NCF laminates under identical VARI conditions, which necessarily produces different fiber volume contents because E-glass rovings compact more efficiently than flax slivers/yarns. The resulting FVCs (~50% for glass vs. ~31% for flax) therefore represent process-realistic outcomes rather than a material property mismatch (Table 8 and Table 9). While RTM and other higher-pressure processes can raise FVC—particularly for flax—our intent here is to report what can be achieved with VARI, as commonly used in industrial settings. Importantly, forcing comparability by reducing glass FVC to match flax would not reflect typical manufacturing practice and would obscure the true process sensitivity of each reinforcement system. In this light, density-normalized metrics help interpret lightweight potential alongside absolute performance, while acknowledging that absolute properties also scale with FVC.

### 4.5. Density-Normalized Performance and Lightweight Potential

When properties are normalized by density, the advantages of the sliver-based architecture become more pronounced. With a composite density of 1.23 g/cm^3^, both flax variants are substantially lighter than the glass fiber reference (1.88 g/cm^3^). The sliver-based flax NCF achieves ~62% of the specific tensile modulus and ~75% of the specific flexural modulus of the glass fiber benchmark, while being roughly one-third lighter.

These findings are consistent with the work of Yan et al. [28] and Zhu et al. [30] who reported that optimized flax laminates can close much of the gap in specific stiffness relative to E-glass, particularly in stiffness-driven applications. This makes the sliver-based flax NCF an attractive option where weight savings are critical—for example, in automotive interior panels, sports equipment, marine components, and architectural cladding.

### 4.6. Implications for Design and Industrial Application

From an engineering design standpoint, the results indicate that the main competitive advantage of the sliver-based flax NCF lies in its stiffness-to-weight ratio rather than in absolute strength. This positions it for semi-structural applications where deformation control is critical but maximum load capacity is less important.

Additionally, the sliver-based NCF are compatible with standard VARI techniques, allowing it to be integrated into existing manufacturing lines with minimal adaptation. This lowers barriers to industrial adoption. However, for more demanding applications, improvements in interfacial adhesion, moisture resistance, and long-term durability will be necessary.

### 4.7. Future Research Directions

The present findings point to several clear pathways for further development:

1. Alternative manufacturing processes—Future work should explore other processing routes, such as Resin Transfer Molding (RTM), which offers the potential to increase fiber volume content by enabling higher compaction pressures and improved resin flow control compared to VARI. Higher fiber volume contents are expected to directly improve stiffness and, in some cases, strength.
2. Impregnation quality assessment—Detailed studies on the quality of resin impregnation are required to ensure uniform fiber wet-out, especially in the more complex architecture of sliver-based NCFs. Special attention should be given to quantifying void content through optical microscopy or micro-computed tomography. The measured porosity should be included in the calculation of composite density to provide more accurate density-normalized mechanical property values.
3. Interface optimization—The fiber–matrix interface remains a key factor limiting strength in flax composites. Future research should investigate surface modification methods, such as silane coupling agents, plasma treatment, or enzymatic processing, to improve adhesion and stress transfer between flax fibers and the matrix. Such improvements could help translate the stiffness gains achieved through optimized fiber alignment in sliver-based NCFs into corresponding strength gains.
4. Durability and aging studies—Long-term performance under realistic service conditions must be evaluated. This includes testing under cyclic loading, elevated humidity, temperature fluctuations, and UV exposure. These studies will help define suitable application environments and inform protective measures such as coatings or barrier layers.

## Figures and Tables

**Table 1:** Overview of the investigated NCFs.

Property	Roving-Based Glass NCF	Yarn-Based Flax NCF	Sliver-Based Flax NCF
Reinforcing Fiber	E-glass, ~2.6 g/cm^3^	Flax, ~1.4 g/cm^3^	Flax, ~1.4 g/cm^3^
Fiber form	Roving, 300 tex	Yarn, 105 tex	Sliver, 5,000 tex
Stitching yarn	text. PES, 76 dtex	text. PES, 76 dtex	text. PES, 76 dtex
Stitching pattern	Fringe	Fringe	Fringe
Layer configuration	+45°/−45°	+45°/−45°	+45°/−45°
Total areal weight	606 g/m^2^	350 g/m^2^	395 g/m^2^
Product name	HP-B600E	ampliTex 5008	-
Producer/ distributor	HP-Textiles GmbH, Schapen, Germany	Bcomp Ltd., Fribourg, Switzerland	ITA of RWTH, Aachen, Germany

**Table 2:** Overview of modules and their respective functions of the modified NCF machine.

Module	Function
Sliver feeding unit	Continuous, twist-free delivery of flax slivers from a rotating can
False-twist unit	Temporary twisting of the slivers for consolidation during feeding
Weft carrier	Placement of slivers at ±45° angles onto a supporting polyester grid
Stitching unit	Fixation of the layers using polyester stitching yarns
Winding unit	Winding of the finished NCF onto rolls

**Table 3:** Specimen dimensions and test setup parameters used for flexural testing.

Material	Sample Thickness (mm)	Sample Width (mm)	Sample Length (mm)	Support Span (mm)
Roving-based glass NCF	1.85	15.00	55.50	37.00
Yarn-based flax NCF	2.98	15.00	89.40	59.60
Sliver-based flax NCF	3.55	15.00	106.50	71.00

**Table 4:** Average local fiber orientation in NCFs. Adapted from [14].

Material	Average Local Fiber Orientation (°)	Average Absolute Deviation from 45° (°)
Roving-based glass NCF	44.27 ± 2.06	1.43 ± 1.64
Sliver-based flax NCF	43.29 ± 2.36	2.05 ± 2.05
Yarn-based flax NCF	46.45 ± 0.86	1.45 ± 0.86

**Table 5:** Tensile properties of NCF composites. Adapted from [14].

Material	Tensile Strength (MPa)	Tensile Modulus (GPa)
Roving-based glass NCF	527.38 ± 13.59	25.61 ± 2.77
Sliver-based flax NCF	126.47 ± 3.61	10.46 ± 0.37
Yarn-based flax NCF	118.87 ± 6.30	8.26 ± 0.05

**Table 6:** Compressive properties of NCF composites. Adapted from [14].

Material	Compr. Strength (MPa)	Compr. Modulus (GPa)
Roving-based glass NCF	437.62 ± 26.31	26.98 ± 2.21
Sliver-based flax NCF	89.64 ± 3.27	9.76 ± 0.68
Yarn-based flax NCF	89.24 ± 1.87	8.62 ± 0.51

**Table 7:** Flexural properties of NCF composites. Adapted from [14].

Material	Flexural Strength (MPa)	Flexural Modulus (GPa)
Roving-based glass NCF	640.30 ± 7.19	13.31 ± 1.40
Sliver-based flax NCF	148.59 ± 12.01	6.58 ± 1.38
Yarn-based flax NCF	144.19 ± 3.46	5.35 ± 0.30

**Table 8:** Fiber volume content, fiber and matrix densities, and resulting composite densities of the investigated NCF laminates. Differences in FVC result from identical VARI processing conditions and thus reflect process-driven compaction behavior.

Property	Roving-Based Glass NCF	Yarn-Based Flax NCF	Sliver-Based Flax NCF
FVC (Vol.-%)	50.03	31.33	31.20
Fiber density (g/cm^3^)	~2.60	~1.40	~1.40
Matrix density (g/cm^3^)	~1.15	~1.15	~1.15
Composite density (g/cm^3^)	1.88	1.23	1.23

**Table 9:** Specific mechanical properties (strength and modulus values normalized by composite density) of the investigated NCF laminates. Adapted from [14].

Material	Spec. Tensile Strength (MPa·cm^3^/g)	Spec. Tensile Modulus (GPa·cm^3^/g)	Spec. Compr. Strength (MPa·cm^3^/g)	Spec. Compr. Modulus (GPa·cm^3^/g)	Spec. Flexural Strength (MPa·cm^3^/g)	Spec. Flexural Modulus (GPa·cm^3^/g)
Roving-based glass NCF	281.20 ± 7.25	13.66 ± 0.32	233.34 ± 14.03	14.39 ± 1.18	341.41 ± 3.83	7.10 ± 0.75
Sliver-based flax NCF	102.99 ± 2.94	8.52 ± 0.30	73.00 ± 2.66	7.95 ± 0.55	121.00 ± 9.78	5.36 ± 1.12
Yarn-based flax NCF	96.77 ± 5.13	6.73 ± 0.04	72.65 ± 1.52	7.02 ± 0.42	117.38 ± 2.82	4.36 ± 0.24

## Data Availability

The experimental work and data presented in this study were generated as part of a doctoral thesis at RWTH Aachen University, 2024 [14]. Several tables and figures have been adapted and translated from this thesis to ensure accessibility in English and consistency with the journal style. All relevant data are included in the article; further inquiries can be directed to the corresponding author.
